# Generation of human myogenic progenitors from pluripotent stem cells for in vivo regeneration

**DOI:** 10.1007/s00018-022-04434-8

**Published:** 2022-07-08

**Authors:** Hyunkee Kim, Rita C. R. Perlingeiro

**Affiliations:** Lillehei Heart Institute, Department of Medicine, University of Minnesota, 4-128 CCRB, 2231 6th St. SE, Minneapolis, MN 55455 USA

**Keywords:** Pluripotent stem cells, Human myogenic progenitors, Transgene-free, Transgene-dependent, In vivo regeneration, Muscular dystrophy

## Abstract

Muscular dystrophy encompasses a large number of heterogeneous genetic disorders characterized by progressive and devastating muscle wasting. Cell-based replacement strategies aimed at promoting skeletal muscle regeneration represent a candidate therapeutic approach to treat muscular dystrophies. Due to the difficulties of obtaining large numbers of stem cells from a muscle biopsy as well as expanding these in vitro, pluripotent stem cells (PSCs) represent an attractive cell source for the generation of myogenic progenitors, given that PSCs can repeatedly produce large amounts of lineage-specific tissue, representing an unlimited source of cells for therapy. In this review, we focus on the progress to date on different methods for the generation of human PSC-derived myogenic progenitor cells, their regenerative capabilities upon transplantation, their potential for allogeneic and autologous transplantation, as well as the specific challenges to be considered for future therapeutic applications.

## Introduction

Muscle degeneration affects millions of individuals globally and may result from different pathological conditions, including muscular dystrophies (MD), sarcopenia, cachexia, metabolic disorders, and chronic muscle injuries. MD in particular refers to an incurable group of inheritable genetic diseases characterized by progressive skeletal muscle weakness leading to paralysis and eventually death due to cardiorespiratory insufficiency [1]. MD is caused by mutations in muscle-related genes that lead to the dysfunction of essential proteins affecting myofiber integrity, and ultimately, muscle fiber viability and muscle contraction [2–4]. At present, more than 40 different types of genetic mutations have been identified in association with different types of MDs [5]. The severity of muscle damage, the age of onset, gender, and the affected muscle groups vary among these disorders [4, 6]. A systematic review reported a prevalence of 16.14 per 100,000 for total combined muscular dystrophies [7]. Among these, the most prevalent and severe is Duchenne MD (DMD), an X-linked recessive type of MD affecting 1 in 5000 male live births. Although there has been much progress in the understanding of disease pathogenesis and testing of novel therapeutics, at present, there is still no cure for DMD or any other type of MD [8]. Most current therapeutic development has focused on gene therapy [9], but strategies aimed at replacing diseased muscle tissue with skeletal muscle stem/progenitor cells able to give rise to healthy functional muscle and self-renew are also promising.

The first proof-of-concept studies for cell-mediated muscle regeneration in DMD focused on the transplantation of myoblasts, which through cell fusion, led to the development of new or hybrid muscle fibers expressing dystrophin [10–13]. Evaluation indicators for transplantation efficiency in these early experimental studies relied solely on the assessment of dystrophin expression levels by immunofluorescence staining and western blot assays. Clinical trials in DMD patients also included measurement of strength in treated muscles [14–17]. Despite positive outcomes in terms of dystrophin expression in early transplantation studies in mdx mice [10–13], no improvement was reported in DMD patients enrolled in early phase clinical trials [16–19], which was overall attributed to limited survival and migratory capacity of injected cells [13, 18, 20]. Instead of myoblasts, their precursor, the muscle stem cells (also known as satellite cells) would be preferable for cell transplantation since these cells, characterized by the expression of the transcription factor Pax7 [21], have the ability to contribute to skeletal muscle regeneration as well as self-renew. A major issue is that in vitro satellite cell expansion would be required for therapeutic applications due to the unfeasibility of harvesting sufficient numbers of satellite cells without permanently damaging the donor muscle. However, ex vivo expansion results in the loss of their intrinsic engraftment capacity as satellite cells rapidly differentiate into myoblasts [22, 23]. Several studies have reported conditions that promote the in vitro expansion of satellite cells [24–30], but these have not yet enabled clinical translation of a satellite cell-based therapy.

This constraint led many investigators to seek alternate types of progenitors for muscle regeneration [31]. Among these, pluripotent stem cells (PSCs) are particularly attractive due to their unlimited proliferative capacity, ability to generate multiple cell types, including skeletal muscle, and amenability to genetic modifications. In this review, we will focus on the different methodologies reported so far for the generation of engraftable human muscle from PSCs, their advantages and disadvantages, and important aspects to be taken in consideration for potential future therapeutic application.

## PSCs and their therapeutic potential

PSCs are characterized by unlimited in vitro expansion potential and the ability to differentiate into virtually all cell types of the body. PSCs encompass embryonic stem cells (ESCs) and induced PSCs (iPSCs). Human ESCs were first isolated from the inner cell mass of early-stage blastocysts by James Thomson’s group in 1998 [32], and since then, numerous studies have documented the generation of therapeutic lineage-specific cell types from these cells [33]. Almost a decade later, Shinya Yamanaka and colleagues reported the generation of iPSCs from somatic cells by the forced expression of a cocktail of transcription factors (Oct3/4, Sox2, Klf4, and c-Myc). This technology allows for the generation of patient-specific iPSCs, enabling autologous cell transplantation [34, 35]. Many studies have reported that iPSCs display the same pluripotent features of ESCs [34–41], thus bringing a PSC-based therapy much closer to clinical application since it eliminates the ethical and immunological issues associated with ESCs. There are several ongoing clinical trials involving both allogeneic and autologous transplantation of PSC-derivatives for several diseases, including macular degeneration, Parkinson’s disease, as well as solid tumors and hematological disorders, among others, and so far results prove evidence for safety, supporting the use of PSC-derivatives for clinical application [42, 43].

## Differentiation of human PSCs into myogenic progenitors

**Fig. 1:**
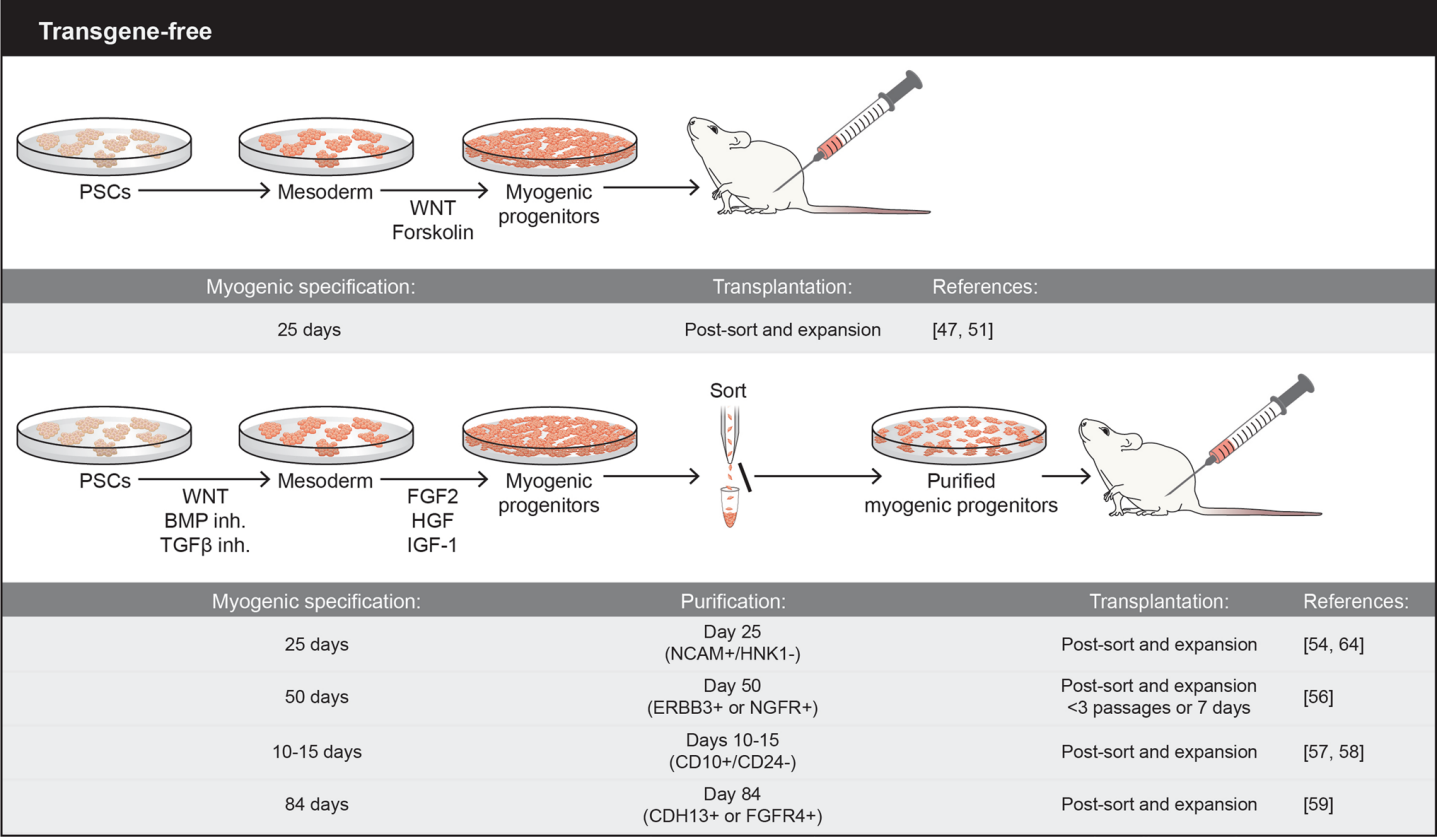
Schematic representation of overall transgene-free methodologies used to generate PSC-derived myogenic progenitors. Major experimental details are depicted

**Fig. 2:**
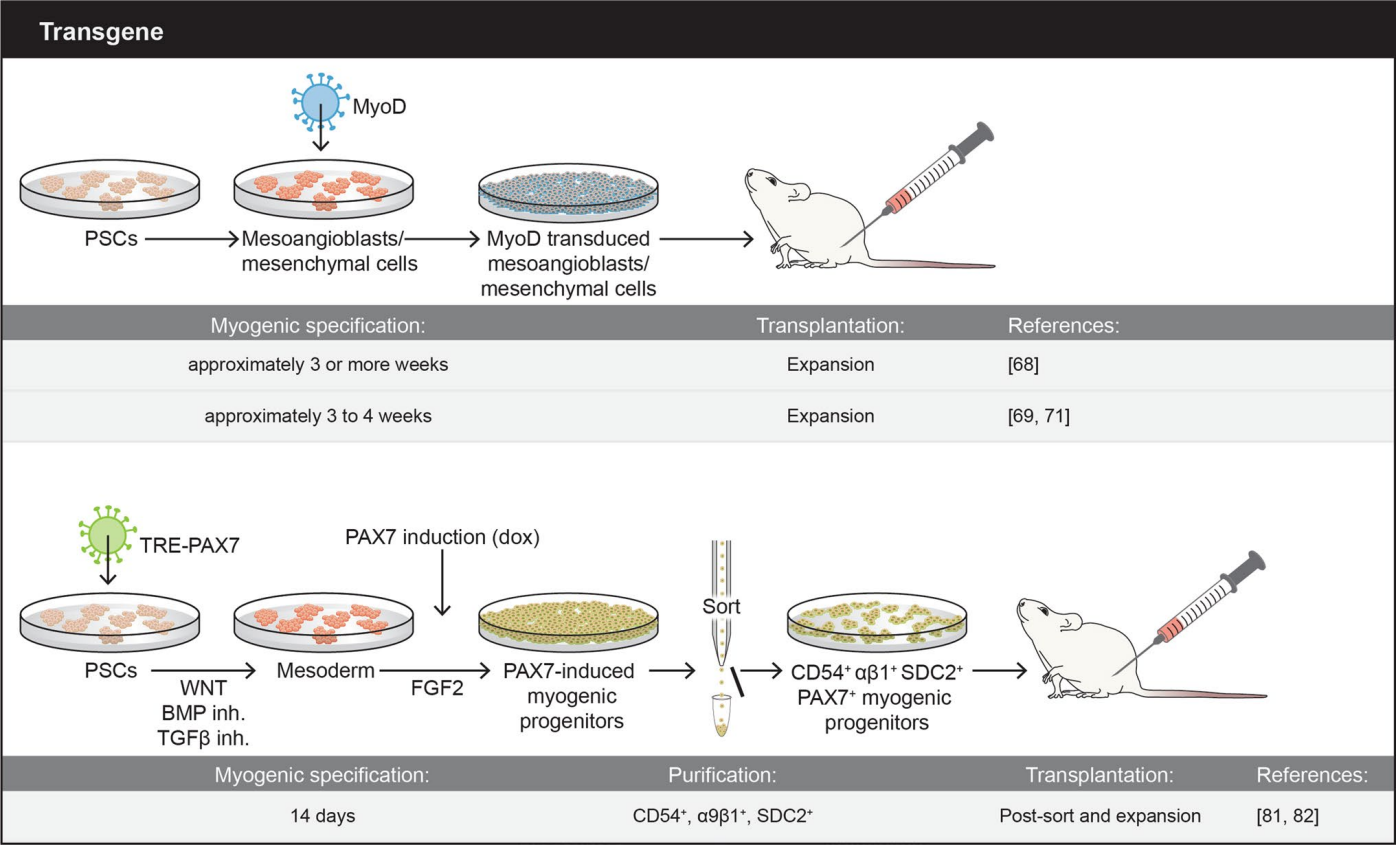
Schematic representation of overall transgene-dependent methodologies used to generate PSC-derived myogenic progenitors. Major experimental details are depicted

To date, skeletal muscle engraftment has been reported upon the transplantation of human myogenic progenitor cells derived from PSCs using several different protocols, including transgene-free, which makes use of defined small molecules (Fig. 1), and transgene-dependent, which utilize overexpression of critical transcription factors of the skeletal muscle hierarchy, such as PAX7 or MyoD (Fig. 2). An important aspect for any given protocol is the ability to generate large numbers of myogenic progenitor cells endowed with significant regenerative potential. Ideally, candidate cells would also seed the satellite stem cell compartment to ensure long-term regeneration. Below, we provide an overview of these protocols, focusing primarily on those that have assessed in vivo regeneration potential.

### Transgene-free protocols

First studies led by Barberi and colleagues in 2007 [44] reported the detection of cells expressing human-specific laminin and nuclear antigen upon the transplantation of 5 × 10^5^ human ESC-derived mesenchymal/myoblast progenitor cells into cardiotoxin (CTX)-injured muscles of SCID/Beige immunodeficient mice. In these studies, target cells were purified on day 35 of differentiation based on the expression of CD73 and NCAM, and subsequently labeled with luciferase, allowing for noinvasive long-term monitoring of engraftment, which was detectable up to 177 days post-transplantation [44]. Five years later, another group reported muscle engraftment upon the transplantation of day 49 unsorted myogenic mesenchymal cells derived from human ESCs and iPSCs (1 or 5 × 10^5^) into irradiated and CTX-injured muscles of NOG mice. Nuclear expression of human LAMIN A/C was detected within myofibers as well as co-localized with Pax7 under the basal lamina, suggesting satellite cell engraftment [45].

Although encouraging, reported engraftment levels were very low, triggering investigators to optimize culture conditions beyond the use of fetal bovine serum (FBS), horse serum (HS) and basic suplementation, such as nonessential amino acids and 2-mercaptoethanol. These efforts led to the use of small molecules to activate WNT signaling by inhibiting glycogen synthase kinase 3 beta (GSK3β) in the initial steps of differentiation, which is critical for optimal induction of paraxial mesoderm (also referred as premyogenic mesoderm) [46, 47]. Subsequent treatment of cultures with fibroblast growth factor 2 (FGF2) enhanced myogenic differentiation [46–50]. Xu and colleagues used a combination of the GSK3β inhibitor BIO, FGF2, and forskolin, and transplantation of resulting day 14 iPSC-derived myogenic cells at 1 × 10^5^ into CTX-injured muscles of NSG mice resulted in the presence of myofibers expressing human δ-Sarcoglycan protein, as well as cells co-expressing Pax7 and human specific histone H2A, suggesting satellite cell engraftment [47]. Using a similar protocol but that included HS, another group of investigators reported engraftment upon the intramuscular (1 × 10^6^) and intravenous (2 × 10^6^) injection of day 14 GFP-labeled iPSC-derived myogenic progenitors in uninjured dystrophin-deficient mdx mice (model for DMD) that were treated daily with immunosuppressant Busulfex [51]. Quantification at 8 weeks post-transplantation showed in average 91 and 85 DYS + myofibers in cohorts that received intramuscular (IM) and intravenous transplantation, respectively. Of note, the authors reported unexpected high numbers of DYS positivity in PBS-injected muscle controls (~ 61%), raising caution about the interpretation of engraftment results. Interestingly, such high numbers of revertant fibers were not observed in the non-injected IM controls nor in the PBS controls for the systemic cohort, suggesting the possibility that needle injury leads to greater fiber reversion in the mdx model [51]. It highlights the usefulness of models with lower fiber reversion rates, such as mdx^4cv^.

In 2015, Chal and colleagues reported an enhanced serum-free monolayer protocol using additional cues from development to induce skeletal myogenesis from mouse and human PSCs [52]. In parallel to WNT activation, the authors applied inhibition of bone morphogenetic protein (BMP) signaling to prevent differentiation of PSCs into lateral plate mesoderm. Subsequently, these cultures were exposed to FGF2, hepatocyte growth factor (HGF), and insulin-like growth factor 1 (IGF1), and within 30 days, these cultures contained large numbers of PSC-derived myogenic cells, including PAX7^+^ satellite-like cells and myotubes [52, 53]. The authors did not document the in vivo regenerative potential of human PSC-derived myogenic progenitor cells generated under this protocol, only the mouse-to-mouse counterparts.

In the following year, Choi and colleagues reported that GSK3β inhibition (day 0 to 4) followed by inhibition of Notch signaling using the γ-secretase inhibitor DAPT (day 4 to 12) led to the generation of expandable hPSC-derived myoblasts [54]. Intramuscular transplantation of 1 × 10^6^ to 3 × 10^6^ myoblasts into irradiated (18 Gy) and CTX-injured NOD-Rag1^null^IL2rg^null^ (NRG) mice resulted in donor-derived muscle contribution, as shown by immunostaining using human specific antibodies to LAMIN A/C and LAMININ [54]. A small fraction of human nuclei co-expressing PAX7 was detected under the basal lamina, suggesting satellite cell contribution. The authors also implemented a purification strategy based on the expression of NCAM (also known as CD56), as previously reported [44], and absence of HNK1. Transplantation of NCAM^+^HNK1^−^ myoblasts generated from control and DMD iPSC-derived myoblasts into CTX-injured muscles of NRG and NSG-mdx^4Cv^ (immunodeficient model for DMD) [55] mice resulted in donor-derived myofiber contribution [54]. Because no comparison was made between unsorted and NCAM^+^HNK1^−^ myoblasts, it was not possible to conclude whether this purification strategy enhanced engraftment efficiency.

In 2018, Hicks and colleagues reported a study in which they compared different established directed differentiation protocols to generate myogenic progenitors/myoblasts from PSCs [56]. Specifically, they assessed the in vivo regenerative potential of day 50 PSC-derived myogenic progenitors (1 × 10^6^) generated by the two previously published differentiation protocols [46, 52], side-by-side with primary muscle cells from fetal stage (directly isolated and cultured), in CTX-injured muscles of NSG/mdx mice [56]. Significant engraftment, as measured by the presence of human DYSTROPHIN, was observed only upon the transplantation of directly isolated fetal tissue (about 200 engrafted fibers). This dropped significantly when cultured fetal counterparts were injected (< 25). Transplantation of PSC-derived myogenic cells resulted in the presence of several hundred human cells, as indicated by LAMIN A/C staining, but these did not fuse with the host muscle (< 5 engrafted fibers) [56]. The authors then tested whether enriching for NCAM^+^HNK1^−^ [54] would improve in vivo regenerative outcomes, but very limited muscle engraftment was detected when the NCAM^+^HNK1^−^ cell fraction was transplanted, with no differences to unsorted counterparts [56]. Ablation of recipient’s satellite cells with the use of irradiation also did not improve engraftment results. Using RNA sequencing, the authors then identified that the surface receptors ERBB3 and nerve growth factor receptor (NGFR) enrich for PAX7^+^ fetal muscle progenitor cells as well as PSC-derived myogenic progenitors, including PAX7^+^ [56]. Injection of 1 × 10^6^ ERBB3 + or NGFR + myogenic progenitors into CTX-injured muscles of NSG/mdx mice, resulted in the presence of muscle fibers expressing human-specific LAMIN A/C, SPECTRIN, and DYSTROPHIN [56], which was much more evident in mice that had been transplanted with the ERBB3 + cell fraction and treated for 2 weeks with the TGFβ inhibitor SB-431542 [56]. Of note, the authors detected virtually no muscle engraftment upon the transplantation of NCAM+ cells, with or without SB-431542 treatment [56].

In the same year, taking advantage of a dual PAX7/MYF5 reporter PSC line, Wu and colleagues screened for differentiation conditions that produced optimal generation of PAX7 cells [57]. These studies led to a protocol consisting of a first step in which cells were cultured for 4 days in the presence of CHIR99021 and SB431542 (GSK3β and TGFβ inhibition, respectively), EGF, insulin, dexamethasone, and 5% HS. On day 4, cells were collected and plated in the presence of medium II, containing LDN193189 (BMP inhibition), SB431542, EGF, HGF, FGF-2, IGF-1, 5% HS, and insulin for 6–11 days. At this point, the authors implemented a purification protocol for the enrichment of PSC-derived myogenic progenitors based on the expression of CD10 and absence of CD24 [57]. Upon sorting, these cells were expanded in the same culture pre-purification conditions (medium II) for an additional 2–3 days. Transplantation of 3 × 10^5^ CD10+ CD24- myogenic progenitors derived from multiple PSC lines in CTX-injured muscles of NSG/mdx mice resulted in significant engraftment, as shown by the presence of myofibers co-expressing human LAMIN A/C and DYSTROPHIN. Quantification of human nuclei expressing PAX7 showed about 12% of donor-derived satellite cell engraftment [57]. This study also assessed myofiber engraftment using ERBB3 and NGFR surface markers, but these provided inferior results [57]. Recently the same authors showed that transplantation of PSC-derived CD10^+^CD24^−^ cells in a mouse model of volumetric muscle loss resulted in myofiber and satellite cell engraftment, as well as functional recovery [58]. Another group, using a MYF5 reporter PSC line, identified FGFR4 and CDH13 as candidate markers for the purification of PSC-derived myogenic progenitors [59]. These authors showed superior engraftment results when transplanting 1 × 10^5^ CDH13 + or FGFR4+ cells (day 84) into NSG/mdx mice when compared to CDH13- or FGFR4- counterparts [59]. The authors investigated the expression levels of other documented surface markers, such as ERBB3 and NGFR, and the satellite cell marker CD82, but they did not compare engraftment outcomes.

Besides the protocol developments described above, the Sampaolesi’s group has reported that treatment with a cocktail of miRNAs [60] or valproic acid [61] enhances the skeletal muscle differentiation and engraftment potential of mesodermal iPSC-derived progenitors.

In 2022, two new studies in this field have been published. Guo and colleagues [62] reported the engraftment of several MD iPSC-derived myoblasts, referred as iMyoblasts, generated using commercially available skeletal muscle differentiation reagents and the reserve cell selection [63], upon their transplantation into irradiated and barium chloride injured NSG mice [62]. Sun and colleagues [64], using their previously reported differentiation protocol combining GSK3β and Notch signaling inhibition [54], documented the long-term regenerative potential of iPSC-derived myogenic progenitors sorted using the PAX7::GFP reporter system, as transplantation of these cells into injured muscles of NSG-mdx contributed to muscle fibers and satellite cells, which were reactivated upon reinjury [64].

### Transgene-dependent protocols

Several studies have taken advantage of the well-established hierarchy of transcription factors regulating the skeletal myogenic lineage to induce skeletal muscle specification from mouse and human PSCs. More than 30 years ago, Weintraub and colleagues published seminal studies showing that MYOD overexpression results in the conversion of fibroblasts and other cell types into myoblasts [65–67]. Based on this premise, Goudenege and colleagues developed a two-step protocol to generate myoblasts from human PSCs [68]. The first step consisted of culturing undifferentiated PSCs in the presence of myogenic medium to induce mesenchymal-like differentiation, as previously described [44], then followed by a second step, in which cells were infected with an adenovirus expressing MyoD under the ubiquitous promoter CAG. Transplantation of 5 × 10^5^ PSC-derived MyoD+ myoblasts into muscles of immunodeficient Rag/mdx mice gave rise to myofibers expressing human SPECTRIN (150–200 engrafted fibers), and some of these co-expressed human DYSTROPHIN [68]. In the same year, Tedesco and colleagues used a lentiviral vector encoding tamoxifen-regulated MYOD to promote myogenic differentiation from PSC-derived mesoangioblasts [69], which resulted in donor-derived myofiber contribution (~ 50) upon transplantation of 1 × 10^6^ cells into α-sarcoglycan (SGCA)-null immunodeficient mice [69]. This MYOD inducible system was further utilized for the generation of in vitro 3D artificial muscle constructs, which could be detected upon implantation in muscles of injured immunodeficient mice by staining with LAMIN A/C, with evidence of vascularization [70]. Additional studies also documented the use of MYOD to generate engraftable myogenic progenitors from PSCs [71–73].

Because MYOD is expressed in myoblasts and at the early stage of myotube formation [74, 75], it may confer limited proliferative capacity to PSC-derived myogenic progenitors. An attractive transcription factor to be used for induction of myogenic specification is PAX7, which is upstream of MYOD, and essential for maintenance of the satellite cell pool [75–80]. Using a doxycycline-conditional expression system for PAX7, Darabi and colleagues [81] reported the generation of highly expandable PAX7-induced myogenic progenitors from multiple PSCs, which upon transplantation into NSG-mdx^4Cv^ mice (5 × 10^5^), gave rise to muscle fibers expressing human LAMIN A/C and DYSTROPHIN (~ 100 myofibers) [81]. The highly expandable nature of these PAX7-induced cells under conditional doxycycline enables the scalability that future clinical trials will require, with the possibility of producing billions of PAX7+ progenitors. The authors reported persistent engraftment at 11 months post-transplantation, in agreement with observed repopulation of the satellite cell compartment, as indicated by the presence of cells co-expressing PAX7 and human LAMIN A/C under the basal lamina [81], and improved contractile force in transplanted muscles compared to PBS-injected controls [81]. Using transcriptome analysis, subsequent studies by Magli and colleagues identified CD54, integrin α9β1 and syndecan2 as surface markers able to purify PSC-derived PAX7+ myogenic progenitors by FACS [82]. Transplantation of PAX7+ myogenic progenitors purified based on the expression of these three surface markers contributed to muscle regeneration in NSG-mdx^4Cv^ mice at a similar rate to myogenic progenitors purified based on GFP (PAX7) expression [82]. Over the years, the PAX7-dependent protocol was further optimized [82, 83] to incorporate small molecules shown to enhance paraxial mesoderm (GSK3β inhibition) [46, 47] and specification of the skeletal muscle lineage (inhibition of BMP and TGFβ signaling) [52, 57, 84]. The use of small molecules enhances the efficiency of PAX7-induced myogenic specification and reduces the variability among independent PSC lines, but these do not replace PAX7 induction [85]. In a recent study, Kim and colleagues utilized a doxycycline-inducible PAX7 transgene inserted into the genomic safe harbor locus, AAVS1 [86], for the alternative strategy of lentiviral PAX7 delivery to generate PAX7 + myogenic progenitors [87]. This strategy showed regenerating muscle fibers upon the transplantation into CTX-injured TA muscles of NSG, NSG-mdx4^Cv^, and C3KO-NSG [87]. Other groups have used PAX7 induction to generate human skeletal muscle from PSCs [88, 89], notably Rao and colleagues generated skeletal muscle bundles, which upon implantation into muscles of NSG or nude mice, survived, vascularized and maintained functionality [89].

## Transplantation of gene edited patient-specific iPSC-derived myogenic progenitors

**Fig. 3:**
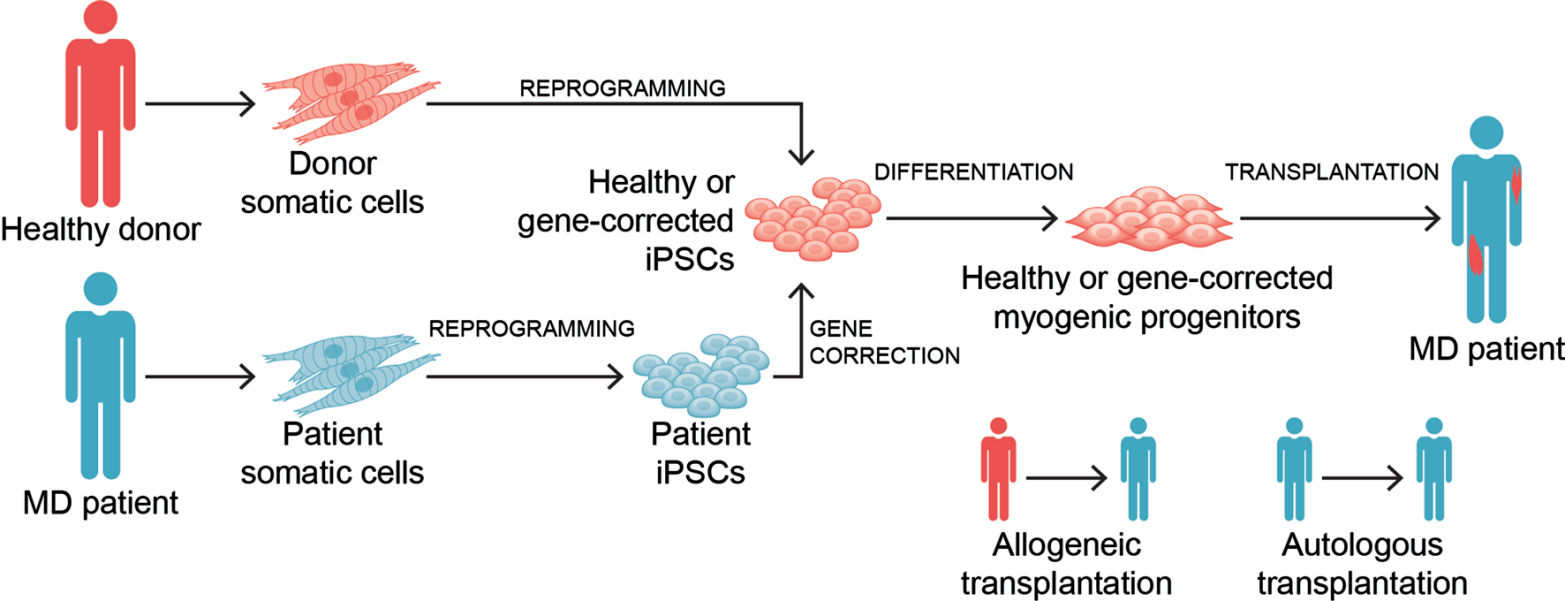
Scheme outlines transplantation modalities for the potential therapeutic application of PSC-derived myogenic progenitors. In the allogeneic setting, somatic cells obtained from a healthy donor are reprogrammed into iPSCs, and following myogenic differentiation, healthy PSC-derived myogenic progenitors are transplanted into MD patients. In the autologous setting, somatic cells obtained from a given MD patient are reprogrammed into iPSCs, and following gene correction and myogenic differentiation, gene corrected PSC-derived myogenic progenitors are transplanted into the MD patient (donor and recipient are the same)

Both allogeneic and autologous cell transplantation have the potential to treat MD patients (Fig. 3). For allogeneic transplantation, one would utilize myogenic progenitors derived from iPSCs derived from a healthy donor (Fig. 3), whereas the autologous approach would generate myogenic progenitors from MD patient-specific iPSCs, and therefore, require in vitro genetic correction prior to transplantation (Fig. 3).

Several studies have documented successful gene correction of MD patient-specific iPSCs, as shown by in vivo rescue following the transplantation of corrected skeletal muscle derivatives, generated using transgene-free, MyoD or PAX7 methodologies.

Gene correction for dystrophin was reported by Young and colleagues [90], who utilized dual gRNAs to knockout DMD exons 45–55 to restore the reading frame in DMD patient-specific iPSCs displaying different types of mutations (deletion mutation of exons 46–51, deletion mutation of exons 46–47, and duplication of exon 50). Since this is considered a mutational hotspot, the authors postulated that this strategy could cover approximately 60% of DMD mutations. In vivo rescue of DYS protein expression was confirmed by the presence of a few donor-derived myofibers expressing human DYS upon the transplantation of gene corrected DMD iPSC-derived myogenic progenitors into NSG/mdx mice [90]. These studies made use of MYOD-ERT lentivirus or the transgene-free approach prior to optimization, which may explain the relatively low number of donor-derived myofibers. Accordingly, engraftment was much superior in the follow-up study [56].

Several studies have reported in vivo rescue upon the transplantation of myogenic progenitors from genetically corrected limb-girdle muscular dystrophy (LGMD) iPSC lines. The first gene correction study was performed in alpha sarcoglycan (SGCA)-mutant iPSCs obtained from patients with LGMD type 2D (LGMD2D/R3) by Tedesco and colleagues using lentiviral delivery of human SGCA cDNA along with the muscle-specific myosin light chain 1F promoter and enhancer [69]. Rescue of α-sarcoglycan gene/protein expression was detected upon IM and intra-arterial transplantation of LGMD2D iPSC-derived SGCA-engineered mesoangioblasts, generated using MyoD overexpression, into SGCA–null immunodeficient mice [69]. In 2019, Selvaraj and colleagues [91] reported the gene correction of Calpain 3 (CAPN3) in iPSC lines obtained from 3 patients with LGMD type 2A (LGMD2A/R1). Since mutations of all three patients were downstream of exon 15, the authors utilized CRISPR/CAS9-mediated homologous recombination to introduce wild-type sequences of CAPN3 ex15-24 cDNA (CAPN3 has 24 exons) at the 3’ end of exon 14. CAPN3 mRNA expression was detected only when CAPN3-null immunodeficient mice were transplanted with gene corrected LGMD2A iPSC-derived PAX7^+^ myogenic progenitors, and not when uncorrected counterparts were injected [91]. More recently, Dhoke and colleagues [92] documented a universal gene correction approach for fukutin-related protein (FKRP), whose mutations are associated with a broad spectrum of muscular dystrophies, including LGMD type 2I (LGMD2I/R9) and the severe Walker-Warburg syndrome (WWS). This strategy replaces the entire FKRP open reading frame with wild-type sequence, thus is able to correct virtually all mutations within the FKRP gene. Transplantation of immunodeficient FKRP-mutant mice with gene corrected WWS iPSC-derived PAX7^+^ myogenic progenitors resulted in rescue of α-dystroglycan functional glycosylation [92].

## Conclusions

PSC-derived myogenic progenitors hold great promise for the future treatment of MDs. As discussed above, there has been significant progress over the past 10 years on the development and optimization of methodologies to generate skeletal myogenic progenitors from human PSCs. Importantly, there has been consensus that the use of specific small molecules and growth factors enhance the skeletal myogenic specification and differentiation from human PCSs, regardless of whether the protocol is transgene-free or not (Fig. 2). Unfortunately, there has been less unanimity in terms of purification protocols. For transgene-free methodologies, NCAM+ HNK1-, ERBB3+ or NGFR+ , CD10**+ **CD24-, and CDH13 + or FGFR4 + have been reported as optimal purification strategies but to date, none have been reproducibly validated. This may be due to the discrepancy in the timeline for purification and transplantation among different protocols, which varies from approximately 12–84 days. With such large time window, it is likely that the cell population under investigation may not be the same among different studies, which may explain the difficulty in validating purification strategies. The only way to solve this issue would be not only to test a given purification strategy but to reproduce the entire protocol and transplantation procedure to the letter. For the PAX7-dependent protocol, the use of CD54, integrin α9β1 and syndecan2 is tailored to this strategy of generating myogenic progenitors since the above-referred surface markers were identified following doxycycline (PAX7) induction, and therefore, characterize a cell population expressing high levels of PAX7.

Both transgene-free and transgene-dependent protocols have advantages and disadvantages. Advantages for transgene-free protocols include the absence of genetic modification, and the simple and direct application of key molecules to the culture medium to generate PSC-derived myogenic progenitors. Although in early years a common difficulty was the heterogeneous nature of generated cells, optimal culture conditions combined with the utilization of surface markers for purification of the target myogenic cell population is likely to circumvent this problem. This aspect is usually less of an issue with transgene-dependent protocols since exogenous expression of specific transgenes directs the differentiation of PSCs towards the desired myogenic cell population. This combined with purification methodologies in general leads to the generation of more homogeneous cell preparations. Another advantage is the ability to generate large quantities of myogenic progenitors when a conditional expression system is used for overexpressing of a given transgene, which is required when considering clinical application. Although transgene delivery through viral vectors may raise concern of insertional mutagenesis, the development of safe self-inactivating lentiviral vectors [93], which have resulted in successful lentiviral-based gene therapy clinical trials [94] provide evidence of safety. Alternatively, the AAVS1 genomic safe harbor locus can be used for genetic modification [87].

Advances in genome editing techniques have allowed several investigators to correct multiple MD-associated genes in patient-specific iPSCs, with demonstrated rescue of functional protein expression, allowing one to envision autologous cell transplantation, besides the allogeneic cell transplantation option (Fig. 3). Critical aspects for the clinical application of PSC-derived myogenic progenitors are scalability, robust and long-term engraftment, safety, and delivery. Given the significant progress over the last decade, it is reasonable to anticipate that a PSC-based skeletal muscle cell therapy may eventually be translated to MD patients in the near future.

## Data Availability

Not applicable.
